# Prevalence of self-reported symptoms of diabetic autonomic dysfunction in the North Denmark Region: a population-based survey

**DOI:** 10.1007/s00592-024-02390-2

**Published:** 2024-11-06

**Authors:** Maria Bitsch Poulsen, Anne-Marie Wegeberg, Johan Røikjer, Amar Nikontovic, Peter Vestergaard, Christina Brock

**Affiliations:** 1 Mech-Sense, Department of Gastroenterology, Aalborg University Hospital, Aalborg, Denmark; 2 Department of Clinical Medicine, Aalborg University, Aalborg, Denmark; 3 Thisted Research Unit, Aalborg University Hospital Thisted, Thisted, Denmark; 4 Department of Endocrinology, Aalborg University Hospital, Aalborg, Denmark; 5 Steno Diabetes Center North Denmark, Aalborg, Denmark

**Keywords:** Diabetic autonomic neuropathy, Diabetes, Patient-reported outcomes, COMPASS-31

## Abstract

**Aims:**

Diabetic autonomic neuropathy is a severe complication of diabetes, estimated to affect up to 44% in type 1 diabetes (T1D) and 73% in type 2 diabetes (T2D) based on clinical studies. Currently, the assessment of diabetic autonomic neuropathy is not implemented in Denmark’s clinical guidelines, complicating the estimation of the true prevalence. Thus, this study investigated the prevalence of self-reported symptoms of autonomic dysfunction in people living with diabetes in the North Denmark Region using the Composite Autonomic Symptoms Score (COMPASS)-31 questionnaire.

**Methods:**

In 2022, all adults with T1D or T2D in the North Denmark Region (*n* = 29,155) were identified using The National Health Insurance Service Registry and invited to an online survey including the Danish version of COMPASS-31. The prevalence and associated 95% confidence intervals (CI) for symptomatic autonomic dysfunction were determined using a cut-off value of 16.

**Results:**

In total, 7,377 completed COMPASS-31, of which 82.4% reported having T2D and 13.7% T1D. The prevalence of symptomatic autonomic dysfunction was 36.8% (95% CI: 34–40) after a median of 26 years with diabetes for T1D and 44.2% (95% CI: 43–45) after a median of 10 years for T2D. Pupillary and orthostatic intolerance were the most frequent moderate to severe symptoms, respectively (38.4% and 24.0% in T1D and 32.8% and 26.3% in T2D).

**Conclusion:**

Symptoms of autonomic dysfunction are very common in individuals with diabetes living in the North Denmark Region, emphasizing the unmet need for regular testing to increase awareness and allow for adequate management, ultimately reducing the morbidity of diabetes.

## Introduction

Diabetes-related complications are a major cause of morbidity and mortality with multiorgan implications. One of the most wide-reaching and debilitating is diabetic autonomic neuropathy [1, 2]. Yet, it is one of the least recognized and poorly understood complications of diabetes [3]. It affects cardiovascular, gastrointestinal, genitourinary, ocular, and secretory functions, impacting physical function and quality of life [4]. Previous prevalence estimates vary widely due to differences in study design and cohorts [3, 5] with studies reporting 33–44% in T1D [6, 7] and up to 73% in T2D [8]. Autonomic impairment has been observed in prediabetes and newly diagnosed type 2 diabetes (T2D) as well [9, 10]. A major challenge of diabetic autonomic neuropathy is its subclinical stage, which eventually develops into a manifest diagnosis associated with poor prognostic outcomes, increased morbidity, and mortality [11]. Emphasizing the importance of screening for early signs of diabetic autonomic neuropathy as current clinical management consists of controlling risk factors such as hyperglycemia, hypertension, obesity, and dyslipidemia, which can reduce the risk of autonomic neuropathy [12] but has no disease-modifying capacity.

Regular testing for diabetic autonomic neuropathy is recommended in Denmark [13] but has yet to be implemented in clinical practice, meaning that it is likely to be overlooked. Recently, patient-reported outcomes have gained attention, providing an opportunity to assess the symptom burden from a patient’s perspective. The Composite Autonomic Symptom Score (COMPASS-31) is a questionnaire used to assess autonomic function and it is useful in the investigation of symptoms of autonomic dysfunction (SAD), but it is not diagnostic tool. Thus, this study aims to investigate the self-reported prevalence of SAD using the validated questionnaire COMPASS-31 digitally distributed to the population with T1D or T2D in the North Denmark Region.

## Methods

### Study population and data source

This study was based on an online survey distributed in The North Denmark Region (Denmark), which consists of 11 municipalities inhabiting about 10% of the Danish population (~ 600,000 citizens) [14]. In November 2022, the Business Intelligence Unit at Aalborg University Hospital identified all adults with T1D or T2D in the region using The Danish National Health Insurance Service Register. This registry links social security numbers to invoices from general practitioners to the Regional Health Administration. Individuals were identified by the International Classification of Diseases 10th Revision (ICD-10) codes DE101-109 and DE110-119 or by chronic care services linked to T2D (0131 or 0132) without the termination code (0133) to exclude those terminated from this service. The identified individuals were invited to the survey hosted on REDCap (Research Electronic Data Capture, Vanderbilt University, USA) [15, 16] via Digital Post, the national IT solution for secure e-mail communication between authorities and citizens. REDCap is a secure electronic data capture system hosted in the North Denmark Region. The survey was open from November 2022 to February 2023. For reference, the same survey was available from February to October 2023 for non-diabetic controls.

## Survey information

The survey included multiple questionnaires, and this paper presents the results from the validated Danish version of the COMPASS-31 [17]. COMPASS-31 is an internally consistent, abbreviated version of the Autonomic Symptom Profile [18]. It comprises 31 questions covering seven domains of the autonomic nervous system: orthostatic intolerance, vasomotor, secretomotor, gastrointestinal, bladder, and pupillomotor function. It is scored from 0 to 100, with higher scores indicating more impaired function. It is validated for use in diabetes against cardiovascular autonomic neuropathy [19–23]. The COMPASS-31 score was determined using Sletten’s method [18], and symptoms were categorized as moderate or severe if they scored within the upper two-thirds (33–100%) of the maximum domain score, thereby excluding occasional mild discomfort comparable to that of non-diabetic individuals. The collected demographical data included age, sex (female/male/other), body mass index (BMI), disease duration, and anti-diabetic medication, as well as socioeconomic parameters: education, employment status, income before tax, and municipality. Long-term diabetes was defined as a duration of ≥ 10 years, and income levels were converted to the income categories: low, middle, and high, using the 33rd and 67th percentiles as thresholds.

### Statistical analysis

Descriptive statistics were expressed according to the data distribution evaluated using QQ plots, histograms, and Shapiro-Wilks tests. Using the total COMPASS-31 score the cohort was stratified according to the severity of the symptoms into SAD- (< 16), SAD+ (16–31), and SAD++ (≥ 32). Different cut-off values exist for diabetic autonomic neuropathy [19–21], and in our laboratory, we use a score of ≥ 16 to define autonomic dysfunction to ensure early autonomic dysfunction is included [24]. Prevalence estimates were given as percentages with confidence intervals (CI) determined by the exact method for binomial distributions. Proportional differences were determined with chi-square tests, and non-normally continuous distributed data are compared using Kruskal-Wallis test with Benjamini-Hochberg correction. Spearman’s rank correlation assessed continuous variables, and logistic regression determined odds ratios for binary outcomes. Analyses were performed in Stata (StataCorp. 2023. Stata Statistical Software: Release 18. College Station, TX: StataCorp LLC) with a significance level of 0.05 and 95% CIs. Figures were produced in R (R Core Team (2023). R: A Language and Environment for Statistical Computing. R Foundation for Statistical Computing, Vienna, Austria) using the ggplot2 and forestplot package.

## Results

### Response rate

Out of 29,155 identified with T1D or T2D identified, 5,949 (20%) did not use Digital Post, leaving 23,206 for invitation. A total of 9,913 respondents replied, with 2,192 (22.1%) excluded for reasons such as duplicates, being non-diabetic, declining participation, incomplete demographic information, other diabetes types, or non-regional residents. After excluding 344 respondents who did not complete COMPASS-31, 7,377 responses were included for analysis. From the non-diabetic participants, 164 (40.9%) declined participation, had diabetes, non-regional residents, or did not complete demographic information or COMPASS-31. Therefore, 299 responses were included for comparison.

**Fig. 1:**
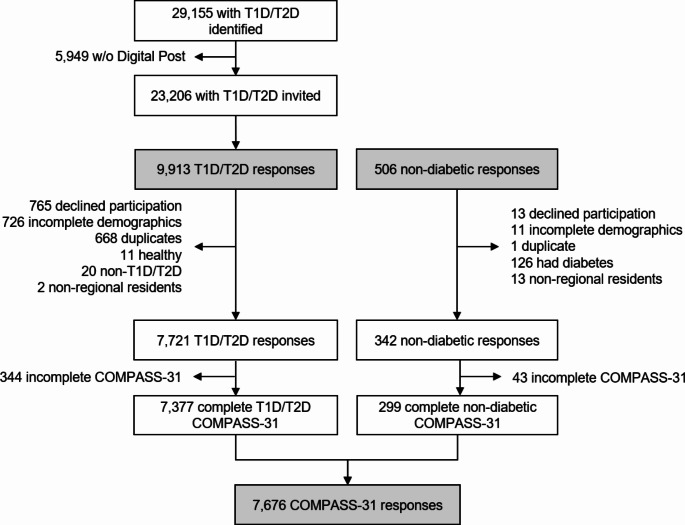
Flowchart of inclusion. COMPASS-31: Composite Autonomic Symptom Score. T1D: type 1 diabetes, T2D: type 2 diabetes, w/o: without

### Clinical characteristics

The groups differed in age, sex, BMI, education, and income. The non-diabetic controls were older than people with T1D (*P* < 0.001) but younger than those with T2D (*P* < 0.001). The T2D was older than the T1D (*P* < 0.001). People with T2D had the highest BMI (vs. non-diabetic: *P* < 0.001, vs. T1D: *P* < 0.001). The non-diabetic group had a higher proportion of individuals with higher education (*P* < 0.001), high-income levels (*P* < 0.001) as wells as females (*P* < 0.001) compared to the diabetes groups. The median COMPASS-31 score was 6.7 [2.8–16.8] for the non-diabetic, 8.9 [3.0-22.5] for people with T1D, and 12.5 [5.3–26.4] for people with T2D. Thus, the non-diabetic had significantly lower scores than people with T1D (*P* < 0.01) and T2D (*P* < 0.001), while people with T2D scored higher than people with T1D (*P* < 0.001). The most reported symptoms were orthostatic intolerance and pupillary problems (Table 1).

**Table 1:** Clinical characteristics

	Non-diabetic	Type 1 Diabetes	Type 2 Diabetes
n (%)	299 (3.9)	1,049 (13.7)	6,328 (82.4)
Age, median (IQR)	62 (53; 70)	59 (47; 68)	67 (59; 74)
BMI, median (IQR)	26.0 (23.6; 29.4)	25.7 (23.2; 29.0)	29.5 (26.2; 33.6)
Sex, n (%)			
Female	195 (65.2)	465 (44.3)	2,466 (39.0)
Male	104 (34.8)	580 (55.3)	3,848 (60.8)
Other	0 (0.0)	< 5 (.)	14 (0.2)
Disease duration, median (IQR)		26 (12; 40)	10 (4; 15)
Education, n (%)			
Municipal primary/lower secondary school (10 years)	32 (13.6)	230 (25.4)	1,978 (35.1)
Upper secondary school/Academy profession graduate (11–15 years)	19 (8.1)	97 (10.7)	472 (8.4)
University graduate, BSc/MSc (16 ≥ years)	185 (78.4)	579 (63.9)	3,178 (56.5)
Income, n (%)			
Low	47 (15.7)	244 (23.3)	1,756 (27.7)
Middle	91 (30.4)	318 (30.3)	2,044 (32.3)
High	105 (35.1)	266 (25.4)	1,105 (17.5)
Unknown	56 (18.7)	221 (21.1)	1,423 (22.5)
COMPASS-31 total, median (IQR)	6.8 (2.8; 16.9)	8.9 (3.0; 22.5)	12.5 (5.3; 26.4)
Moderate/severe symptoms, % (95% CI)			
Orthostatic intolerance	14.4 (10.6–18.9)	24.0 (21.5–26.7)	26.3 (25.2–27.4)
Secretomotor	5 (2.8–8.1)	11.2 (9.3–13.2)	10.3 (9.5–11.0)
Vasomotor	8.7 (5.8–12.5)	12.3 (10.3–14.4)	15.8 (14.9–16.7)
Gastrointestinal	7.4 (4.7–10.9)	9.3 (7.6–11.2)	12.3 (11.5–13.2)
Bladder	7.7 (4.9–11.3)	12.4 (10.5–14.6)	19.8 (18.8–20.8)
Pupillary	36.5 (31.0-42.2)	38.4 (35.5–41.4)	32.8 (31.7–34.0)

IQR: interquartile range, BMI: body mass index, CI: confidence interval

## Prevalence of autonomic dysfunction symptoms

**Fig. 2:**
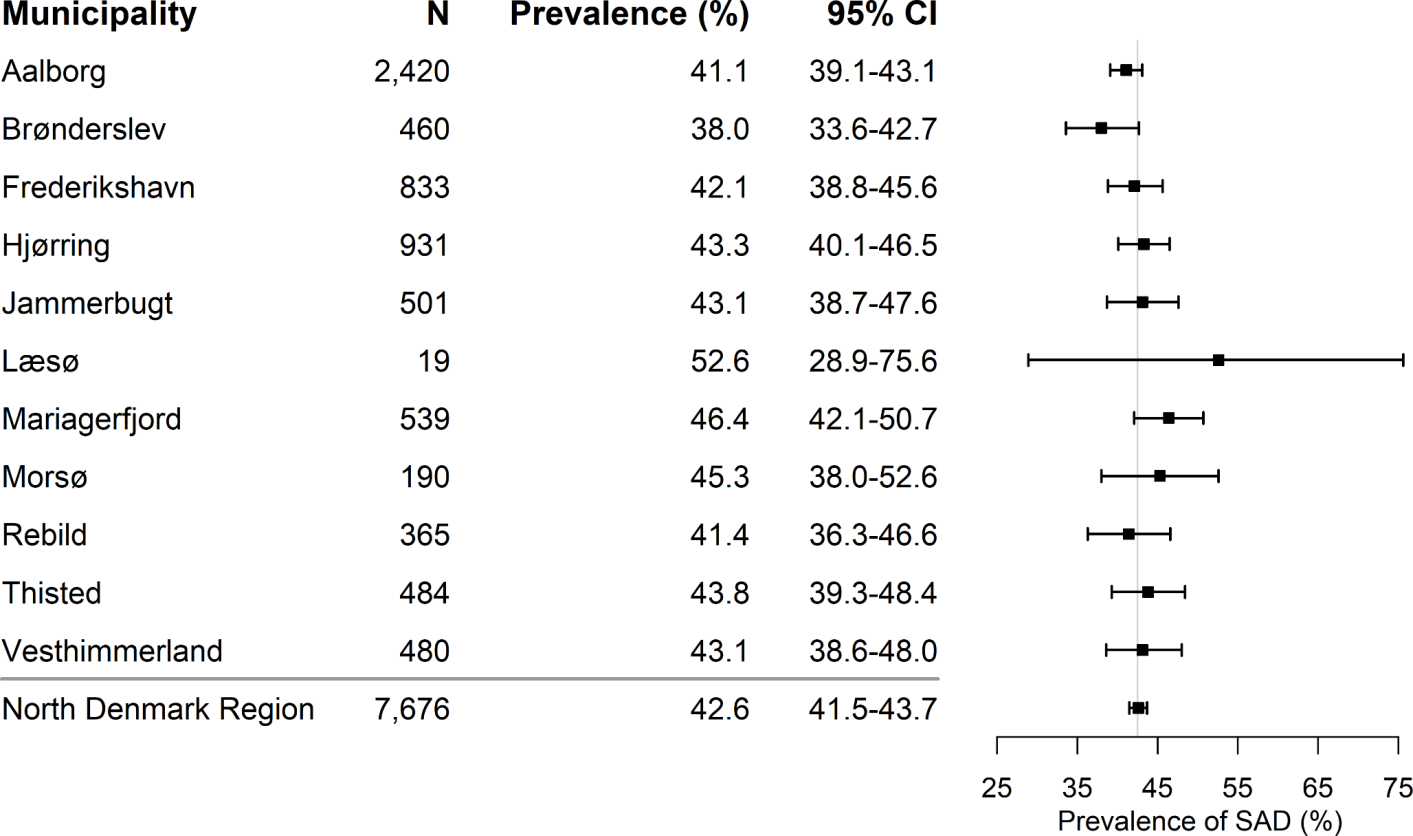
Geographical distribution of symptoms of autonomic dysfunction in the North Denmark Region. CI: confidence interval. SAD: symptoms of autonomic dysfunction

The prevalence of SAD was 36.8% (CI: 34–40) in people with T1D, 44.2% (CI: 43–45) in people with T2D, and 29.1% (CI: 24–34) in the non-diabetics. SAD was more prevalent in both T1D and T2D compared to the non-diabetics (*P* < 0.05, *P* < 0.001) and more common in T2D than in T1D (*P* < 0.001). The prevalence of SAD ranged from 38.0 to 52.6% in the 11 municipalities, with a regional level of 42.6% and the CIs from all municipalities overlap with those of the regional level (Fig. 2).

## Severity of autonomic symptoms

**Fig. 3:**
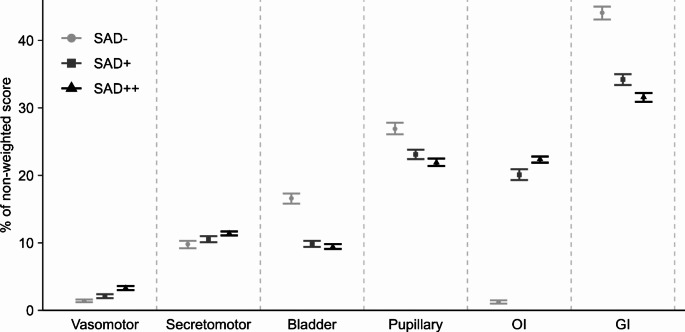
Interval plot of the non-weighted domain scores’ contribution to the total non-weighted score expressed as mean ± confidence intervals. OI: orthostatic intolerance, GI: gastrointestinal

In the analysis of the stratified groups, significant differences were found in age (*P* < 0.01), BMI (*P* < 0.001), disease duration (*P* < 0.05), and educational level (*P* < 0.05). However, these differences were small and had no clinical relevance even though there was statistical significance, which is attributed to the large sample. A higher proportion of low-income and fewer high-income households were observed in SAD++ (*P* < 0.001) (Table 2). The groupwise comparison showed that the non-diabetic with SAD was better educated with higher incomes compared to those with diabetes and SAD (*P* < 0.01, *P* < 0.01). In the domain analysis, gastrointestinal symptoms contributed most to the score, followed by pupillary symptoms when assessing the non-weighted score. Interestingly, orthostatic intolerance contributes ~20% in SAD (SAD + and SAD++) compared to only 1.2% in those without SAD (SAD-) (Fig. 3).

**Table 2:** Clinical characteristics comparing different stages of diabetic autonomic neuropathy

	SAD-	SAD+	SAD++
n (%)	4,408 (57.4)	1,876 (24.4)	1,392 (18.1)
Age, median (IQR)	66 (58; 73)	65 (57; 73)	65 (57; 73)
BMI, median (IQR)	28.4 (25.2; 32.1)	29.3 (25.8; 33.5)	30.0 (26.3; 34.3)
Diabetes duration, median (IQR)	10 (5; 18)	10 (5; 17)	10 (5; 20)
Diabetes type, n (%)			
Non-diabetic	212 (4.8)	63 (3.4)	24 (1.7)
Type 1 Diabetes	663 (15.0)	220 (11.7)	166 (11.9)
Type 2 Diabetes	3,533 (80.1)	1,593 (84.9)	1,202 (86.4)
Education, n (%)			
Municipal primary/lower secondary school (10 years)	1,299 (33.6)	503 (30.4)	438 (35.1)
Upper secondary school/Academy profession graduate (11–15 years)	330 (8.5)	161 (9.7)	97 (7.8)
University graduate, BSc/MSc (16 ≥ years)	2,241 (57.9)	988 (59.8)	713 (57.1)
Income, n (%)			
Low	1,030 (23.4)	528 (28.1)	489 (35.1)
Middle	1,459 (33.1)	573 (30.5)	421 (30.2)
High	894 (20.3)	388 (20.7)	194 (13.9)
Unknown	1,025 (23.3)	387 (20.6)	288 (20.7)

SAD: symptoms of autonomic dysfunction, IQR: interquartile range. BMI: body mass index

### Risk factors for symptoms of autonomic dysfunction

**Fig. 4:**
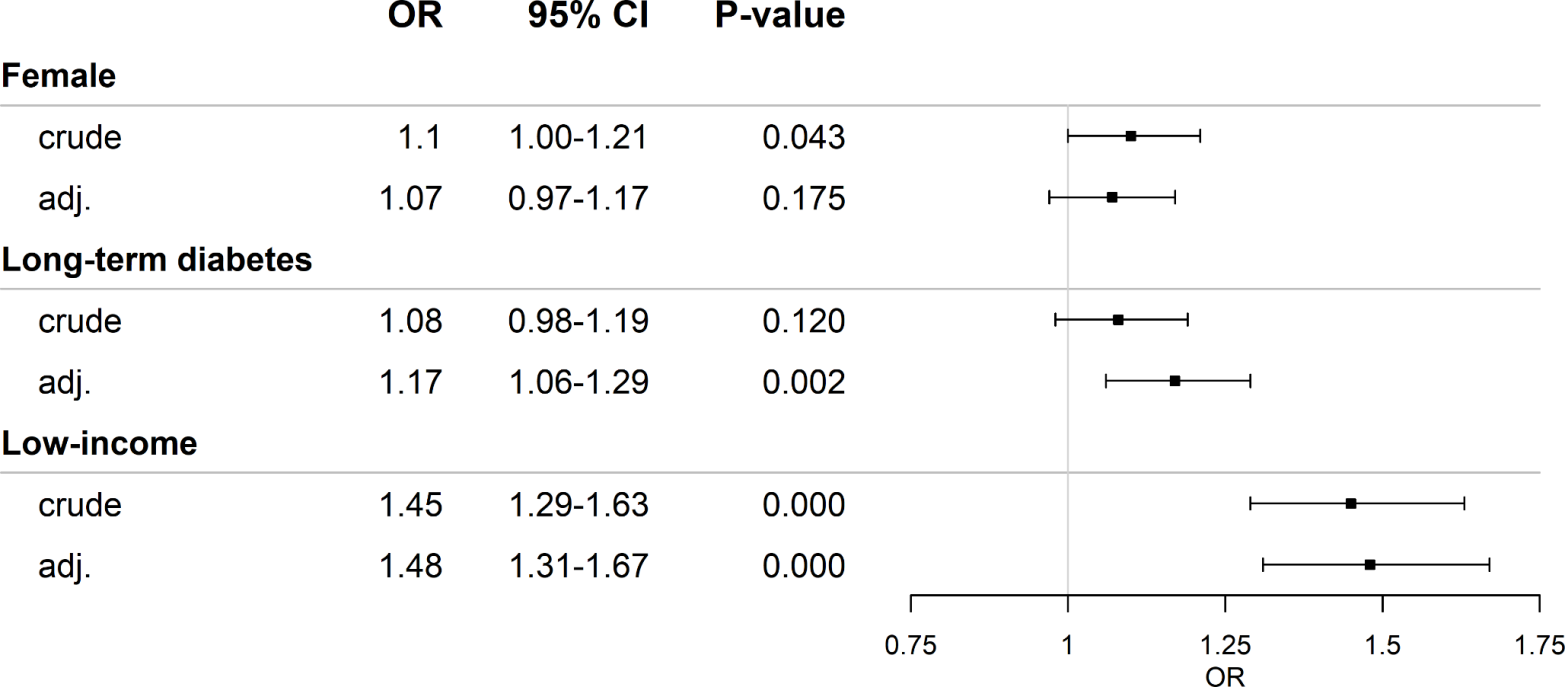
Risk factors for symptoms of autonomic dysfunction. Female and long-term diabetes was adjusted for age and BMI. Low-income was adjusted for age, BMI, and retirement. OR: odds ratio, adj.: adjusted, CI: confidence interval

Long-term diabetes (≥ 10 years) increased the odds for SAD by 17% when adjusted for age and BMI (1.17 [1.06–1.29], *P* < 0.01), while low income increased the odds by 48% when adjusted for age, BMI, and retirement (1.48 [1.31–1.67], *P* < 0.001). Females had higher COMPASS-31 scores than males across all groups (non-diabetic: 8.24 (3.34; 18.02) vs. 5.42 (2; 12.12), T1D: 10.10 (3.45; 23.98) vs. 8.71 (2.89–21.33), T2D:13.20 (6.24; 27.56) vs. 11.93 (4.79; 25.75)), but did not have higher odds for SAD when adjusted for BMI and age (Fig. 4). Moreover, a weak positive association between the score and the diabetes duration (*P* < 0.05, ρ = 0.02) and a weak negative association between the total score and age (*P* < 0.05, ρ = 0.03) was observed. The large sample size drove the statistical significance, but the clinical significance of the relationship is minimal. Lastly, insulin, glucagon-like peptide-1-receptor (GLP-1) agonists or sodium-glucose co-transporter-2 (SGLT2) inhibitor-users had higher COMPASS-31 score than non-users (insulin: 17.4 (7.9; 26) vs. 11.5 (4.7; 25), GLP-1: 16.3 (7.4;29.6) vs. 11.3 (4.5; 24.9), SGLT2-inhibitor: 14.1 (6.2; 27.8) vs. 11.7 (4.7; 25.6), all *P* < 0.01), but only insulin and GLP-1 users had a median score ≥ 16. No difference in metformin or dipeptidyl peptidase-4 (DPP-4) inhibitors DDP4 (*P* > 0.05) was observed.

## Discussion

This regional survey showed that the symptomatic prevalence of SAD is 37% in people with T1D after 26 years of disease and 44% in people with T2D after 10 years, compared to 29% in the reference non-diabetic group. Orthostatic intolerance and pupillary symptoms were the most common, and low-income was the most important risk factor for SAD. No major geographical differences were observed. Additionally, increasing COMPASS-31 scores were not associated to disease duration or age, demonstrating the silent presentation and the unmet clinical need for regular testing of autonomic dysfunction.

One study estimated the prevalence of SAD to be 17% in T1D with a disease duration of 27 years using the Survey of Autonomic Symptoms [25]. This finding is considerably lower than that of this study, probably due to differences in the populations. Mainly because the T1D Exchange Clinic Registry is a voluntary online community in the US, likely featuring more compliant participants with fewer complications than the general diabetic population. In contrast, this study identified eligible participants from the National Health Insurance Service Registry, providing a broader population sampling. To our knowledge, no other exclusively symptom-based prevalence estimations of SAD have been conducted in T2D and the ongoing DD2 study does not include autonomic measures [26]. Thus, no direct comparator is currently available for the T2D estimate in this study. Most estimations, based on the gold standard cardiovascular autonomic reflex tests, range from 33 to 73% [6–8], and these are likely higher due to the inclusion of the subclinical stages of SAD. Yet, this study shows that even when the subclinical stages are omitted, there is still a relatively high prevalence, reflecting the underrecognition of SAD in the clinical practice in Denmark. Moreover, the non-diabetics presented as expectedly with the lowest prevalence of SAD. However, the relatively high prevalence of 29% can be explained by the complex pathophysiology of autonomic dysfunction. It can be caused by many different disorders, but due to the nature of this study, we unfortunately do not have any information on the presence of other causes of autonomic dysfunction than diabetes in this group.

The geographical range of SAD varied from 38 to 52%, with Læsø being the biggest outlier. Læsø is the municipality with the fewest inhabitants in this region, and with only 19 responses, this estimate may be uncertain. However, as an island municipality with limited access to specialized care, the higher prevalence of SAD may reflect increased expenses and travel-time required for adequate care. In contrast, Brønderslev had the lowest prevalence, which could be an indicator of a well-functioning primary sector with high interdisciplinary collaboration. In general, no major geographical differences in SAD were observed within this region, indicating that urban and rural citizens across the region receive similar healthcare services.

In terms of COMPASS-31 scores, the group with T2D presented with higher scores than those with T1D, consistent with Low (2004), where the Autonomic Symptom Profile was used [8]. In the T1D Exchange Study dry mouth/eyes were one of the most common symptoms [25]. Nonetheless, in this study, these symptoms were also common in the non-diabetic control group. However, pupillary disturbances causing light sensitivity in individuals with diabetes are plausibly related to SAD. Symptoms of orthostatic intolerance are caused by a failing sympathetic nervous system, i.e., a late-stage marker of SAD, and it constituted ~ 20% of the non-weighted score in those with SAD (≥ 16). This could suggest a lower cut-off value to identify early diabetic autonomic neuropathy. In the same line, diagnosis of orthostatic intolerance is easily performed measuring the blood pressure response from lying to standing and could serve as a surrogate marker for severe SAD.

Moreover, gastrointestinal symptoms had the greatest impact on the score, in agreement with Melling 2023 [27], emphasizing the importance to recognize the gastrointestinal symptom burden in diabetes. These symptoms were more common in people with T2D (12.3%) than in people with T1D (9.3%), which may be linked to the use of GLP-1 agonists, known to possess gastrointestinal adverse reactions. This is supported by the observation that GLP-1 users had higher COMPASS-31 scores. Additionally, insulin and SGLT2-inhibitor-users also had higher COMPASS-31 scores, but these are not first-line treatments for T2D suggesting a longer disease duration and potentially poorer glycemic control contributing to the COMPASS-31 score and not necessarily the medication on its own.

We found no clinically meaningful relationship between increasing COMPASS-31 scores and disease duration or age, and disease duration has previously been found to be a poor predictor of cardiovascular autonomic neuropathy as well [28]. Long-term diabetes did increase the odds for SAD, which emphasizes that the introduction of improved diabetic medication has delayed the onset of complications, plausibly due to reduced hypo and hyperglycemic events. Nonetheless, regular control of autonomic functions is relevant in diabetes regardless of age and disease duration for early detection to decrease the morbidity and mortality of diabetic autonomic neuropathy particularly from major cardiovascular events [29, 30].

Regarding quality of life, females are known to report poorer physical health compared to men on a global scale [31], and this study showed that they tended towards higher COMPASS-31 scores as well. Nevertheless, being female did not increase the odds of developing SAD meaning the higher proportion of females in the non-diabetic group did not falsely increase the prevalence estimate in this group. In terms of socioeconomics, this study showed that lower educational levels were associated with both T1D and T2D. In a previous Danish register study, similar results were shown for T2D [32]. This association might be mediated by co-morbidities, e.g. overweight, hypertension and dyslipidemia in T2D, whereas the early onset of T1D can impact educational attainment due to the challenges of managing a chronic condition during crucial developmental years. In comparison to a previous regional socioeconomic study, the individuals of this study had a larger proportion of individuals with a higher educational level [33]. This study associated low-income with both T1D, T2D, SAD severity, and identified it as a major risk factor for SAD, highlighting a socioeconomic dimension to diabetic complications in agreement with the T1D Exchange study [25]. Low-income has previously been associated with increased health care risk [34] that may be mediated by, e.g. diminished understanding of health, reduced capacity to engage in physical activities, fewer resources to utilize the free public health care services or even mistrust of the healthcare system. Thus, targeted intervention aimed at the low-income segment could be emphasized to prevent diabetic complications.

An advantage of this study is the application of the Danish National registries, The National Health Insurance Service Registry combined with the patient-reported symptom burden. We used the register to identify citizens with diabetes, as the quality and coverage in this registry are considered very high, because it is based on the invoices required for reimbursement of the general practitioners from the Regional Health Administration. However, the validity of the registry has not been confirmed [35]. The shortcoming is the risk of false diagnosis to increase reimbursement to the general practitioner. However, only < 0.1% answered that they did not have diabetes. Consequently, it is unlikely that individuals with diabetes and access to Digital Post were not invited to the survey. Those without Digital Post were excluded from the survey, constituting ~ 20% of the regional population with diabetes compared to only 5% nationwide [36]. Exemption criteria can be physical or mental disabilities or lack of access to digital devices, etc. Thus, it may be speculated that those without Digital Post more often are vulnerable patients likely to have complications. A 4x higher exemption rate in the population with diabetes indicates substantial under-reported complications, potentially greatly underestimating the prevalence of SAD. In addition, the voluntary and comprehensive nature of the survey may have skewed our dataset by omitting people with fewer resources.

One of the limitations of patient-reported outcomes is the lack of a clinical confirmation of the diagnosis. In general, an epidemiological study of the relevant ICD-10 codes for diabetic neuropathies from the Danish health registries is preferred. However, until testing for diabetic autonomic neuropathy is implemented in the clinical practice, the Danish registries will not have sufficient information. Therefore, patient-reported outcomes are the best alternative at this point. It should be added that when symptoms are used as indicators of diabetic autonomic neuropathy, the subclinical stages will be omitted; hence, the estimate will be an underestimation of the true prevalence. Moreover, this survey was entirely based on questionnaires thus, information on co-morbidities and concomitant medication was not accessible. Therefore, it was not possible to exclude symptomatic overlap with other diseases independent of diabetes, e.g., allergies, functional gastrointestinal burdens, which may have overestimated the prevalence. In addition, no data on glycemic control was available since this paper is based exclusively on self-reported data and glycated hemoglobin levels would then be vulnerable to recall bias without validation. Lastly, even though Denmark is a small country with free health care, regional differences exist, and the Danish Diabetes Association found that the highest proportions of individuals with glycated hemoglobin > 70 mmol/mol live in the North Denmark Region [37]. For that reason, it is plausible that the prevalence is higher in this region and, thus, may not be entirely generalizable at the national level. However, this study provides a digital platform that can be easily applied nationally and to other countries with similar digital access to citizens.

In conclusion, SAD are common among individuals with T1D and T2D in the North Denmark Region. The identification of the patient-experienced burden and risk factors could aid targeted prevention and treatment strategies to improve quality of life and health care expenditures. This study highlights that there is an unmet need for regular testing of autonomic neuropathy to increase awareness and allow for adequate management of diabetic autonomic neuropathy.
